# Ecological factors affecting first-time mothers’ satisfaction with *Sanhujoriwons* (postpartum care centres) from South Korea: a cross-sectional and correlational study

**DOI:** 10.1186/s12884-023-05770-8

**Published:** 2023-06-20

**Authors:** Ju-Eun Song, Soyeon Lee, Min Kyong Lee, Hyun-Ju Chae

**Affiliations:** 1 College of Nursing & Research Institute of Nursing Science, Ajou University, Suwon, Republic of Korea; 2 Department of Nursing, Ajou University Medical Center, Suwon, Republic of Korea; 3 Department of Nursing, Samsung Medical Center, Seoul, Republic of Korea; 4 Department of Nursing, Joongbu University, 201, Daehak-ro, Chubu-myeon, Geumsan-gun, Chungnam, 32713 Republic of Korea

**Keywords:** Postnatal care, Personal satisfaction, Partnership, Social support, Ecology

## Abstract

**Background:**

In South Korea, commercial postpartum care centres, known as *Sanhujoriwon*s, have emerged as important institutions aiding mothers’ physical recovery after childbirth. Although previous studies have measured mothers’ satisfaction level with *Sanhujoriwon*s, this study applies Bronfenbrenner’s ecological model to identify the factors influencing first-time mothers’ satisfaction with *Sanhujoriwons*.

**Methods:**

This descriptive correlational study involved 212 first-time mothers admitted to *Sanhujoriwon*s for two weeks with their new-borns (healthy babies weighing at least 2.5 kg) after giving birth after 37 weeks of pregnancy. Data were collected using a self-report questionnaire at five postpartum care centres in the metropolitan area of South Korea from October to December 2021, on the day of the mothers’ discharge. This study considered ecological factors such as perceived health status, postpartum depression, childcare stress, maternal identity at the individual level; partnership with *Sanhujoriwon* staff at the microsystem level; and the *Sanhujoriwon*s’ education support system at the exo-system level. The data were analysed using descriptive statistics, t-test, one-way ANOVA, correlation analysis, and hierarchical regression analysis using the SPSS 25.0 Win program.

**Results:**

The mean score of satisfaction with *Sanhujoriwons* was 59.67 ± 10.14 out of 70, indicating a high level of satisfaction. The hierarchical regression analysis showed that satisfaction with *Sanhujoriwons* was significantly affected by the perceived health status (β = 0.19, *p* < 0.001), partnership between mothers and the caregivers (β = 0.26, *p* < 0.001), and education support system of the *Sanhujoriwons* (β = 0.47, *p* < 0.001). The explanatory power of the model for these variables was 62.3%.

**Conclusions:**

Our results indicate that not only the mother’s health status but also the educational support system of postpartum care centres and partnerships are important for improving first-time mothers’ satisfaction with postpartum care centres. Thus, when developing an intervention program for postpartum care centres, practitioners should focus on developing various kinds of support and strategies to improve the physical health condition of mothers, build partnerships between mothers and care staff, and improve the quality of the educational support offered to mothers. Further studies to develop and test the effectiveness of such intervention programs are strongly suggested.

## Background

*Sanhujori* is a Korean cultural concept of postpartum care that emphasizes the restoration of a woman who has given birth to her pre-pregnancy healthy state [1]. In Asian societies, postpartum care has traditionally been provided by family members at homes [2]. However, as the family structure has shifted from extended to nuclear families, new mothers often lack the required family support for postpartum care [1, 2]. Consequently, many institutions providing postpartum care services, called *Sanhujoriwons*, have appeared in South Korea [1]. *Sanhujoriwons* are typically non-medical commercial institutions; since the first one was established in 1996, their utilization has continued to increase [3]. In 2021, 81.2% of mothers who gave birth in South Korea had utilized a *Sanhujoriwon* [1]; therefore, using a *Sanhujoriwon* after childbirth seems to have become a key representative of the culture of childbirth care in South Korea.

*Sanhujoriwon*s have developed and implemented various programs for postpartum care [1], and staff and facilities are managed in compliance with the legal standards of the Mother and Child Health Act [4]. However, a 2018 study [3] reported that mothers who used *Sanhujoriwon* were less satisfied with its services, and satisfaction with the postpartum care provided at *Sanhujoriwon* was lower than that provided at their parental homes [1]. Mothers’ satisfaction with care is a crucial variable for evaluating the quality of care provided [5–8]. Therefore, it is necessary to consider whether *Sanhujoriwon* provides appropriate postpartum care.

The average duration of stay at a *Sanhujoriwon* is approximately 2 weeks [1]. After delivery, mothers stay in the hospital for 3–7 days and are subsequently admitted to a *Sanhujoriwon* after discharge. Depending on the hospitalisation period, the period of stay in *Sanhujoriwon* is typically three weeks after childbirth. This period of three weeks is important for the mother’s postpartum recovery and also the formation of attachment and the acquisition of the maternal role through interaction between the mother and baby [9]. Previous studies have reported that women who are satisfied with the postpartum care they receive have higher parenting efficacy, start breastfeeding earlier and more effectively, and experience fewer health-related problems within themselves and their babies [10–12]. However, during this three-week period, the lack of appropriate postpartum care services at *Sanhujoriwon* could significantly affect the mother’s health recovery, attachment formation, and maternal role acquisition. Therefore, identifying the factors contributing toward low satisfaction among mothers who use *Sanhujoriwons* is an important step to understand the appropriate measures required to improve *Sanhujoriwons’* services [3]. However, thus far, most studies have only identified the mothers’ satisfaction levels after using a *Sanhujoriwon*, without delving into the factors that influence their satisfaction levels.

Satisfaction with *Sanhujoriwon* is an evaluation criterion for measuring the quality of services received by the mother while interacting with the staff members and the environment of the care centres during their stay at the *Sanhujoriwon*. Women’s experiences with care services have many determinants [13]. In addition, it is well-recognized that satisfaction is a multiplex and multidimensional concept and is affected by numerous elements [14, 15]. Considering this complexity, an ecological model can be applied to identify the factors affecting satisfaction with *Sanhujoriwons*. In the ecological model, individuals are described as beings who develop through interactions with their surrounding environments, which includes micro- (the individual’s immediate environment), meso- (interaction with two or more systems), exo-system (the larger social system), and macro-systems (the larger cultural context) [16]. Based on this ecological model, this study examines the factors influencing mothers’ satisfaction with *Sanhujoriwons*, by dividing them into individual (perceived health status, childcare stress, maternal identity, and postpartum depression), micro-system (partnership between mothers and the *Sanhujoriwon* staff), and exo-system (education support system of a *Sanhujoriwon*) factors.

Among the individual factors influencing mothers’ satisfaction with *Sanhujoriwons*, perceived health status is especially important, as postpartum physical recovery is the main concern driving mothers to use *Sanhujoriwon* [3]. In addition, we include maternal identity and childcare stress as factors because several mothers have reported that performing the maternal role based on the *Sanhujoriwon’s* prescribed programs interfered with their rest time essential for their physical recovery after childbirth [17]. Maternal identity and childcare stress are associated with maternal-role acquisition [18, 19], and mothers with a high maternal identity feel more positively about performing their maternal roles in *Sanhujoriwons* and experience low parenting stress; the reverse is true for mothers with a low maternal identity. In addition, in the two- to three-week period after childbirth, when mothers typically use a *Sanhujoriwon*, they are highly likely to experience postpartum depression [20]; therefore, postpartum depression was included as a variable. A previous study on mothers who used a *Sanhujoriwon’s* services also reported experiencing negative emotions including postpartum depression during their stay [17], which can affect their satisfaction with *Sanhujoriwons*.

Partnership is emphasised as an important factor in child nursing [21, 22]; partnership between parents and nurses implies that the parents actively fulfil their childcare responsibilities, and the nurses help the parents as partners to provide optimal care for children [23, 24]. Partnerships between mothers and nurses, which includes the mother’s interaction with their surrounding human environment, can significantly affect mothers’ perceptions of nursing quality [25]. Furthermore, the time when mothers use a *Sanhujoriwon* is a crucial period for acquiring the maternal role for their postpartum recovery; thus, partnership-formation between the *Sanhujoriwon* staff and the mother is critical. However, studies on partnerships between mothers using a *Sanhujoriwon* and *Sanhujoriwon* staff are rare. Therefore, this study includes mother– *Sanhujoriwon* staff partnership as a micro-system factor. Nevertheless, it excludes husbands and peer mothers who were admitted to the *Sanhujoriwon* during the same period, even though they are important agents who interact with mothers during their stay at the *Sanhujoriwon*. This is because, during the time of this study, owing to COVID-19, the husbands’ stay at the *Sanhujoriwon* and the mothers’ interaction with other mothers were limited.

The educational support system of the *Sanhujoriwons* is a key an environmental feature that can affect mothers admitted at the *Sanhujoriwons*. These mothers have a high demand for childcare-related education [1, 3]. A qualitative study on mothers who used the services offered by postpartum care centres reported that mothers negatively perceived their experience in these centres as they did not provide them with adequate professional education related to childcare [17]. Therefore, we included the educational support system provided by postpartum care centres as an exo-system factor that could affect mothers’ satisfaction levels.

This study aims to examine the extent to which certain individual-, meso-, and exo-level factors affect mothers’ satisfaction with *Sanhujoriwons* during their stay and suggests ways to develop an efficient and professional *Sanhujoriwon* program to improve these mothers’ service satisfaction.

## Methods

### Design

This descriptive and correlational study uses a cross-sectional research design and a self-report questionnaire for data collection and analysis.

### Participants

Initially, between October and December 2021, 258 participants were recruited through convenience sampling from five *Sanhujoriwons* in Seoul and Gyeonggi provinces, South Korea. These five *Sanhujoriwons* are similar in facility size, mother and newborn care, education support systems, and the number of nursing staff within the metropolitan. Participants were included if they: (1) were primiparous women, (2) delivered a healthy infant weighing over 2,500 g after 37 weeks of gestation, (3) were admitted to a *Sanhujoriwon* with their infants, (4) stayed in the *Sanhujoriwon* for two weeks postpartum, and (5) voluntarily agreed to participate in this study. First-time mothers who delivered twins or had health problems including infection, bleeding, or major depression in the postpartum period that needed medical treatment in the *Sanhujoriwon* were excluded. Among the initial participants, 46 were excluded because of missing data; thus, finally, data of 212 participants were analysed. When calculating the post-hoc power analysis of regression using the G*Power 3.1 program, the sample size of 212 reached a power of 99.9%, with an α level of 0.05, conventional medium effect size of 0.15, and seven independent variables [26].

### Measurement

The participants filled out a self-report questionnaire that included satisfaction with the *Sanhujoriwons* as a dependent variable, and independent variables pertaining to the satisfaction with *Sanhujoriwons*, nested in the ecological model—perceived health status, childcare stress, maternal identity, postpartum depression (individual system); partnership between mother and care staff (microsystem); and education support system (exo-system). The measurement tools are as follows:

***Satisfaction with Sanhujoriwons*** was measured through a ‘Scale for assessing satisfaction of *Sanhujoriwon* service’, which was developed by the research team based on the national standard guideline for evaluating *Sanhujoriwons* in South Korea [27] and a previous study exploring mothers’ experience of using a *Sanhujoriwon*’s services [17]. This scale has seven items with a 10-point numerical rating scale (0–10), resulting in a score from 0 to 70; higher scores indicate higher levels of satisfaction with *Sanhujoriwons*. The appropriateness of the scale was reviewed and validated by three experts who had extensive experience with the evaluation criteria development procedure in national institutions and substantial research and practical expertise in postpartum care centres. The Cronbach’s alpha was 0.85.

***Perceived health status*** refers to the mothers’ subjective assessment of their health status and was measured using the one-item Short Form Health Survey using a 10-point numerical rating scale developed by Stewart et al. [28] and translated into Korean by Son et al. [29]. A score of zero means ‘I don’t feel healthy at all’, and a score of 10 means ‘I feel that I couldn’t be any healthier’. Scores range from 0 to 10; higher scores indicate higher level of perceived health status. The 10-point single item numerical rating scale is widely used and has been validated to measure subjective feelings in not only an international study [30] but also a Korean study [29]. In this study, three experts on women’s health confirmed the appropriateness of the scale for postpartum women.

***Childcare stress*** was measured using the Childcare Stress Inventory developed by Cutrona [31], translated into Korean by Cheon [32], and revised by Song [33]. This inventory consists of 14-items rated on a five-point Likert scale (1–5), resulting in a score from 14 to 70; higher scores indicate a higher degree of childcare stress perceived by the mother. A previous study has already established the internal consistency reliability (Cronbach’s alpha = 0.87) and content validity for the use of this scale on postpartum Korean women [34]. In this study, the Cronbach’s alpha was 0.85.

***Maternal identity*** was measured using the Semantic Differential Scale-Myself as Mother, developed by Walker [35] and translated into Korean by Koh [36]. This inventory consists of 11-items rated on a seven-point Likert scale (1–7), resulting in a score from 11 to 77. Higher scores indicate a positive maternal self-identity. Content validity and internal consistency reliability of Cronbach’s alpha = 0.81-0.83 were established in the postpartum women in Korea [36]. In this study, the internal consistency reliability based on Cronbach’s alpha was 0.85.

***Postpartum depression*** was measured using the Edinburgh Postnatal Depression Scale developed by Cox, Holden, and Sagovsky [37] and translated into Korean by Kim [38]. This scale consists of 10-items rated on a four-point Likert scale (0–3), with higher scores indicating a higher degree of depression. Content validity and internal consistency reliability of Cronbach’s alpha = 0.79 were established on postpartum women in Korea [34]. This study also showed good internal consistency reliability (Cronbach’s alpha = 0.82) for this instrument.

***Partnership*** was measured using a partnership measurement tool developed by our research team based on the Partnership Care Delivery Model suggested by Wiggins [39]. This instrument comprises 30-items rated on a five-point Likert scale (1–5), resulting in the score from 30 to 150, with higher scores indicating a higher degree of partnership between the mothers and care staff of *Sanhujoriwons*. The content validity of this instrument was tested by ten experts on childbirth and postpartum care from research and clinical fields. The item level of this scale’s content validity index (I-CVI) was from 0.80 to 1.00, and the scale level of the CVI (S-CVI) was 0.95. The reliability of this scale also showed good internal consistency (Cronbach’s alpha = 0.96) in this study.

***Education support system*** was measured using a scale on perceived education support system developed by our research team based on the national standard guideline for evaluating *Sanhujoriwons* in South Korea [27]. This scale consists of 18 items reflecting the educational content on postpartum and newborn care recommended for postpartum care centres. The content of the scale was reviewed and validated by three experts who have extensive experience with the evaluation criteria development procedure in national institutions and substantial research and practical expertise in postpartum care centres. In this study, the internal consistency of the scale was 0.97.

### Data collection and ethical considerations

Data were collected from five postpartum centres located in the metropolitan areas of South Korea from October to December 2021. First, we explained the purpose and importance of this study to the relevant administrators and team managers of each centre to obtain their cooperation. Thereafter, detailed information on the research purpose and process and guarantees of anonymity were given to the participants who met the inclusion and exclusion criteria. Only those who provided written informed consent to participate voluntarily were given the questionnaire, and they were informed that they could resign from participation and quit answering the questionnaire at any time without any disadvantage. It took the participants approximately 15 min to complete the questionnaire. All participants were given small gifts as rewards for their participation in the study.

This study was reviewed and approved by the Institutional Review Board (IRB) (No. *******-SUR-2021-438) in A hospital before data collection, and all study processes were conducted according to the guidelines and regulations of the Committee on Publication Ethics [40].

### Data analysis

The data were analysed using SPSS version 25.0 (SPSS Inc., Chicago, IL, USA). Descriptive statistics were used to define participants’ demographic characteristics and study variables. An independent sample t-test, analysis of variance, and Scheffe’s test were conducted to identify differences in participants’ satisfaction with *Sanhujoriwons* in accordance with their general characteristics. Pearson’s correlation coefficients were calculated to identify the relationship between satisfaction with *Sanhujoriwons* and the independent variables. Finally, to examine the factors affecting satisfaction with *Sanhujoriwons*, a hierarchical multiple regression analysis was performed.

## Results

### Demographic and obstetric characteristics

The average age of the participants was 32.74 years, and the average rooming-in time for mothers and their babies to stay together in the same room was 4.67 h per day. The majority of mothers were living with their husbands (97.6%), university graduates (79.6%), employed (75.1%), from the middle economic class (67.1%), and had given birth via caesarean Sect. (60.5%). Regarding feeding status, almost one-third of mothers were mostly breastfeeding (39.6%), and one-third were mostly formula feeding (34.0%). A total of 107 participants (50.7%) delivered a boy and the others (104 participants, 49.3%) delivered a girl (Table 1).

**Table 1:** Differences in the satisfaction with *Sanhujoriwon* based on the general characteristics (N = 212)

Characteristics	Categories	n (%)^*^	Mean ± SD	t or F	*p* (scheffe)
Age (years) (32.74 ± 3.65)	< 35 35	155 (73.1) 57 (26.9)	59.86 ± 10.09 59.16 ± 10.34	0.44	0.658
Family type	Couple only With parents	206 (97.6) 5 (2.4)	59.76 ± 10.11 53.80 ± 10.80	1.30	0.195
Educational level	High school University Master’s degree	19 (9.0) 168 (79.6) 24 (11.4)	59.47 ± 13.18 59.72 ± 9.87 59.00 ± 9.71	0.06	0.946
Occupation	No Yes	52 (24.9) 157 (75.1)	58.13 ± 12.69 60.09 ± 9.20	-1.03	0.308
Economic Status	Low ^a^ Middle ^b^ High ^c^	25 (11.9) 141 (67.1) 44 (21.0)	56.71 ± 12.19 59.13 ± 10.38 63.16 ± 6.70	4.00	0.020 (a < c)
Religion	Not having Having	129 (61.4) 81 (38.6)	60.02 ± 9.79 58.95 ± 10.75	0.74	0.460
Delivery type	NSVD Caesarean	83 (39.5) 127 (60.5)	59.36 ± 10.84 59.77 ± 9.74	-0.67	0.506
Feeding method	BF more Half and half FF more	84 (39.6) 56 (26.4) 72 (34.0)	61.43 ± 9.82 58.62 ± 11.07 58.43 ± 9.59	2.12	0.122
Baby’s sex	Male Female	107 (50.7) 104 (49.3)	59.22 ± 10.30 60.15 ± 10.05	-0.67	0.506
Rooming-in (hours/day)			4.67 ± 2.19		

NSVD = normal spontaneous vaginal delivery, BF more = breast feeding more than formula feeding, Half and half = half breast feeding and half formula feeding, FF more = formula feeding more than breastfeeding

^*^ Valid percent

### Descriptive statistic of the study variables

The mean score for satisfaction with *Sanhujoriwons* was 59.67 ± 10.14 out of 70, which shows a relatively high level of satisfaction. The mean scores of perceived health status, childcare stress, maternal identity, and postpartum depression were 7.25 ± 2.01 (out of 10), 33.52 ± 7.68 (out of 70), 52.82 ± 8.16 (out of 77), and 7.07 ± 4.56 (out of 30), respectively. The average scores of partnership with the *Sanhnujoriwon* staff and the *Sanhnujoriwon’s* educational system were 124.02 ± 19.13 (out of 150) and 72.96 ± 12.91 (out of 90), respectively (Table 2).

**Table 2:** Descriptive statistics of the study variables (N = 212)

Variables	Possible range of scores	Mean ± SD	Minimum	Maximum
**Dependent Variable**				
Satisfaction with *Sanhujoriwons*	0–70	59.67 ± 10.14	20	70
**Independent Variables**				
Perceived health status	0–10	7.25 ± 2.01	0	10
Childcare stress	14–70	33.52 ± 7.68	14	54
Maternal identity	11–77	52.82 ± 8.16	24	75
Postpartum depression	0–30	7.07 ± 4.56	0	20
Partnership	30–150	124.02 ± 19.13	30	150
Education support system	18–90	72.96 ± 12.91	40	90

### Differences in the participants’ satisfaction with *Sanhujoriwons* based on their general characteristics

We found a significant difference in the participants’ satisfaction with *Sanhujoriwons* by economic status (F = 4.00, *p* = 0.020); that is, the participants who reported having a high economic status were more satisfied with *Sanhujoriwons* than those with low economic status. There were no significant differences based on other general characteristics (Table 1).

### Relationship between satisfaction with *Sanhujoriwons* and the study variables

Satisfaction with *Sanhujoriwon*s was positively correlated with perceived health status (r = .46, *p* < 0.001), maternal identity (r = .26, *p* < 0.001), partnership (r = .66, *p* < 0.001), and educational support system (r = .74, *p* < 0.001), whereas it was negatively correlated with childcare stress (r = − .33, *p* < 0.001) and postpartum depression (r = − .33, *p* < 0.001) (Table 3).

**Table 3:** Correlation coefficients of the study variables (N = 212)

Study variables	Satisfaction with *Sanhujoriwons* r (*p*)
Perceived health status	0.46 (< 0.001)
Childcare stress	− 0.33 (< 0.001)
Maternal identity	0.26 (< 0.001)
Postpartum depression	− 0.33 (< 0.001)
Partnership	0.66 (< 0.001)
Education support system	0.74 (< 0.001)

### Factors influencing satisfaction with *Sanhujoriwons*

A hierarchical multiple regression analysis was performed to verify the influence of the independent variables constructed based on the ecological model. Model 1 verified the influence of economic status as a control variable on the dependent variable, that is, satisfaction with *Sanhujoriwons*. Models 2, 3, and 4 measured the effects of the individual (perceived health status, childcares stress, maternal identity, postpartum depression), micro (partnership with *Sanhujoriwon* staff), and exosystem (education support) variables, respectively, on the dependent variable.

The result of the final Model 4 showed that the participants’ satisfaction with *Sanhujoriwons* was significantly affected by perceived health status at the individual (β = 0.19, *p* < 0.001), partnership at the microsystem (β = 0.26, *p* < 0.001), and the education support system at the exosystem (β = 0.47, *p* < 0.001) levels. These variables explained 62.30% of the variance in the participants’ satisfaction with *Sanhujoriown* use (Table 4).

## Discussion

This study aimed to identify the factors affecting first-time mothers’ satisfaction with *Sanhujoriwons* (postpartum care centres) based on the ecological model, which comprises, individual, microsystem, and exosystem factors.

In this study, perceived health status was a significant individual factor affecting satisfaction with *Sanhujoriwons;* that is, the better the participants’ perceived health status, the higher their satisfaction with *Sanhujoriwons*. This result aligns with that of a previous study, which reported that perceived health status is a determining factor of women’s satisfaction with maternal.

**Table 4:** Factors predicting satisfaction with *Sanhujoriwons* (N = 212)

Factors	Model I					Model II					Model III					Model IV				
	B	SE	β	t	*p*	B	SE	β	t	*p*	B	SE	β	t	*p*	B	SE	β	t	*p*
Constant	58.77	0.77		75.89	< 0.001	48.63	6.77		7.18	0.148	17.48	6.30		2.78	0.006	11.24	5.65		1.99	0.048
High economic status	4.39	1.69	0.18	2.60	0.010	2.75	1.50	0.11	1.82	0.070	0.53	1.25	0.02	0.43	0.670	-0.23	1.11	-0.01	-0.21	0.838
Perceived health status						1.81	0.34	0.36	5.28	< 0.001	1.39	0.28	0.28	4.92	< 0.001	0.94	0.26	0.19	3.64	< 0.001
Childcare stress						-0.17	0.10	-0.13	-1.60	0.110	-0.06	0.08	-0.05	-0.71	0.482	-0.05	0.08	-0.04	-0.61	0.541
Maternal identity						0.08	0.09	0.06	0.93	0.353	-0.02	0.07	-0.02	-0.28	0.778	0.01	0.06	0.01	-0.03	0.973
Postpartum depression						-0.17	0.18	-0.08	-0.98	0.330	-0.12	0.15	-0.05	-0.81	0.418	-0.08	0.13	-0.04	-0.62	0.436
Partnership											0.29	0.03	0.55	10.24	< 0.001	0.14	0.03	0.26	4.18	< 0.001
Education support system																0.37	0.05	0.47	7.44	< 0.001
R^2^	0.031					0.272					0.520					0.623				
Adj. R^2^	0.027					0.254					0.506					0.610				
F(*p*)	6.73 (0.010)					15.21 (< 0.001)					36.61 (< 0.001)					47.68(< 0.001)				

healthcare [13]. The main purpose of postpartum care recognised by mothers was the restoration of their health [1]. The biggest concern for mothers who chose to be admitted at a *Sanhujoriwon* was receiving sufficient rest for postpartum recovery [3]. Therefore, mothers who perceived their health condition as good were satisfied with their postpartum care centre.

Postpartum women may often experience weakness and vulnerability and thus require special care [2, 9, 41]. Therefore, a *Sanhujoriwon’s* environment, systems, and services are designed to prioritise rest for mothers [42] to facilitate their postpartum recovery [17]. Mothers recognised that staying at the *Sanhujoriwon* enabled them to rest sufficiently and promoted their postpartum recovery [17], which increased their satisfaction with the *Sanhujoriwon*. However, we found that the *Sanhujoriwon* mainly focused on providing care for mothers’ postpartum recovery and did not adequately support maternal role attainment [43]. For instance, most mothers admitted at a *Sanhujoriwon* did not want rooming-in for rest, and *Sanhujoriwons* also do not always conduct rooming-in, but only when mothers want it [17]. Consequently, mothers spend most of their time separated from their babies in *Sanhujoriwons*, which causes problems in acquiring maternal roles such as breastfeeding, mother–infant attachment, and new-born care [2, 43]. To address this problem, postpartum care services should aim to enhance the acquisition of maternal roles while also promoting postpartum recovery.

The reason why mothers who use a *Sanhujoriwon*’s services do not want rooming-in is that they often find it difficult to breastfeed or take care of the baby alone while rooming-in [17]. These difficulties are even greater for first-time mothers who lack knowledge and skills related to breastfeeding or baby care [2, 3]. Therefore, when a mother performs maternal roles, such as breastfeeding or rooming-in for the first time, it is necessary for an expert to help the mother rather than letting the mother do it alone. Thereafter, the nursing staff should observe whether the mother is facing any difficulties while performing the maternal roles and provide continuous education and counselling so that they can address these difficulties and continue to perform their maternal role. This requires policy support to supplement experts *Sanhujoriwons*.

In this study, we examined mothers’ partnership with the *Sanhujoriwon* staff as a micro-system factor affecting their satisfaction with *Sanhujoriwons*. As mentioned earlier, previous studies [22, 25] have reported that the better the partnership with nurses, the higher their satisfaction with nursing care and the higher their perception of the nursing quality [25].

The mother–nurse partnership is important because it helps mothers enhance their efficacy in manage and actively cope with their child [44]; thus, the better the mother–nurse partnership, the higher the mother’s ability to manage the child’s condition [45]. Partnerships between family and school nurses, in which parents participate in school healthcare, also had a more positive effect on their children’s healthcare performance [46].

The context of the mother–nurse partnership also applies to the partnership between the mother and *Sanhujoriwon* staff. Postpartum mothers, as primary caregivers, are responsible for providing overall care to their new-born, including breastfeeding, for which acquiring maternal role is critical. Similar to nurses, the *Sanhujoriwon* staff also support mothers in providing the best care for their new-borns. Our results also showed that mothers with better partnerships with *Sanhujoriwon* staff could perform maternal roles better and had higher self-efficacy. Considering that one of the two main reasons for using a *Sanhujoriwon’*s services is the acquisition of child-care-related knowledge and skills [3], better partnerships may have had a positive impact on mothers’ satisfaction with *Sanhujoriwon*.

However, we also found that most mothers who stay at a *Sanhujoriwon* prioritise their postpartum recovery. Therefore, newborn care at *Sanhujoriwons* is mostly provided by *Sanhujoriwon* staff, and mothers play a passive or observer role in new-born care [17, 47]. Some mothers complained about being asked to participate in new-born care, such as breastfeeding or rooming-in at the *Sanhujoriwon* [17]. Therefore, to form a better partnership between *Sanhujoriwon* staff and mothers, improving the awareness of mothers who choose to stay at a *Sanhujoriwon* is critical. To this end, the social awareness of the culture of using a *Sanhujoriwon’s* services should be improved through education along with the promotion of the importance of breastfeeding and early maternal attachment. As *Sanhujoriwons* cannot ignore consumer demands, such an awareness must be built at the national level, such as by distributing promotional materials and producing and broadcasting educational videos [48].

In this study, we examined the *Sanhujoriwon’s* education system as an exo-system factor affecting mothers’ satisfaction with *Sanhujoriwons*; the more positive the evaluation of the *Sanhujoriwon’s* education system, the higher the mothers’ satisfaction with *Sanhujoriwons*. These assumptions are based on the results of a previous research that reported low satisfaction with postpartum care due to low satisfaction with the educational programs provided by *Sanhujoriwon* [3]. In addition, previous studies have also reported that the higher the expert support, the higher the postpartum care satisfaction [49–51].

Postpartum women need more time to get advice from healthcare professionals about their health and parenting [49]. The most important aspect of postpartum care for mothers who used *Sanhujoriwon*’s services was getting sufficient rest and acquiring the relevant knowledge and skills related to childcare [3]. However, many postpartum women reported negative experiences such as the lack of time for staff to provide meaningful support, lack of information about parenting, and inadequate support for breastfeeding [52]. Mothers who used a *Sanhujoriwon’*s services also reported that they did not receive professional education on childcare or professional help when breastfeeding. [17].

Educational programs related to postpartum care and childcare provided by *Sanhujoriwon* staff are generally insufficient [17, 48]. In addition, most educational programs provided are commercial content that is not directly related to postpartum care or childcare. Alternatively, they are provided by non-professionals from companies related to childcare product rather than by healthcare professionals [17]. This not only lowers mothers’ satisfaction with *Sanhujoriwons* but also has a negative impact on postpartum recovery and maternal role acquisition. Therefore, it is necessary to provide educational programs related to postpartum care and childcare for mothers using *Sanhujoriwon*. These educational programs should include practical programs that reflect the needs of postpartum mothers and must be continuously modified and supplemented by reflecting mothers’ feedback on the programs and the latest information [3, 13]. In addition, mothers who stayed at *Sanhujoriwons* were dissatisfied because the education was a unidirectional transfer of simple instructions without real practice and did not involve the fathers. Therefore, various education methods, such as repeated education with practice, should be utilised, with efforts to include the fathers in childcare education [17].

Additionally, studies related to maternal health have reported the importance of the role of healthcare professionals in postpartum care satisfaction. If healthcare professionals improve clinical practice related to maternal health promotion and well-being, better postpartum care can be provided to postpartum women, improving satisfaction with postpartum care [13, 53, 54]. Healthcare professionals can enhance satisfaction with postpartum care by spending more time with mothers and providing advice on new-born care and feeding [49]. Therefore, it is necessary to strengthen the professionalism of the staff in *Sanhujoriwons* and increase the professional support provided to mothers by increasing the number of professionals in *Sanhujoriwon*. For this, *Sanhujoriwon*s could provide refresher education for their staff in *Sanhujoriwon* and involve more experts, such as breastfeeding experts [48].

### Limitations

This study was performed using convenience sampling at five *Sanhujoriwons*. Thus, there were limitations in controlling the effects of confounding variables or excluding sampling errors although we attempted to control the characteristics of facilities or support systems among different *Sanhujoriwons*. Therefore, further studies using a randomized sampling method at the national level are needed. In addition, this study did not include the fathers and peer mothers who stayed in the *Sanhujoriwon* during the same period as the mother and who form an important support system for mothers admitted at a *Sanhujoriwon*. This is because the husband’s residence in the *Sanhujoriwons* and interaction with peer mothers within the *Sanhujoriwons* were restricted owing to COVID-19. Therefore, further research is needed to identify the factors affecting *Sanhujoriwon* satisfaction, including the support received from mothers’ husbands and peer mothers who stayed at *Sanhujoriwon* during the same period as the mother. Finally, although the *Sanhujoriwon* satisfaction scale was developed based on the national standard guideline for evaluating *Sanhujoriwons* in South Korea [27] and validated by three highly qualified experts on postpartum care centres and women’s health, further validation studies are required to accumulate more qualitative evidence on postpartum care centres.

Despite this limitation, this study contributes to the literature by applying the ecological model to identify the factors affecting satisfaction with *Sanhujoriwons*. In addition, it is meaningful in that it was the first to consider partnership between *Sanhujoriwon* staff and mothers and the effect of this partnership on mothers’ satisfaction with *Sanhujoriwons*.

## Conclusion

In this study, we found that the higher the perceived health status (individual factor), the higher the partnership with *Sanhujoriwon* staff (micro-system factor), the more positive the mothers’ evaluation of the *Sanhujoriwon* educational system (exo-system factor), and the higher the mothers’ satisfaction with *Sanhujoriwons*. Therefore, *Sanhujoriwons* must implement various programs to promote mothers’ physical recovery after childbirth and establish an education system based on the needs of first-time mothers. In addition, there is an urgent need to establish a strategy to facilitate mothers and *Sanhujoriwon* staff to form healthy partnerships.

## Data Availability

The datasets used and analysed during the current study are available from the corresponding author upon reasonable request.
